# How the Integration of Telehealth and Coordinated Care Approaches Impact Health Care Service Organization Structure and Ethos: Mixed Methods Study

**DOI:** 10.2196/20282

**Published:** 2020-10-09

**Authors:** Rosemary Davidson, David Ian Barrett, Lorna Rixon, Stanton Newman, ACT Program

**Affiliations:** 1 Institute for Health Research University of Bedfordshire Luton United Kingdom; 2 School of Health and Social Work University of Hull Hull United Kingdom; 3 Centre for Health Services Research City, University of London London United Kingdom; 4 see Acknowledgments

**Keywords:** coordinated care, telehealth, health care organization, staff engagement, staff training

## Abstract

**Background:**

Coordinated care and telehealth services have the potential to deliver quality care to chronically ill patients. They can both reduce the economic burden of chronic care and maximize the delivery of clinical services. Such services require new behaviors, routines, and ways of working to improve health outcomes, administrative efficiency, cost-effectiveness, and user (patient and health professional) experience.

**Objective:**

The aim of this study was to assess how health care organization setup influences the perceptions and experience of service managers and frontline staff during the development and deployment of integrated care with and without telehealth.

**Methods:**

As part of a multinational project exploring the use of coordinated care and telehealth, questionnaires were sent to service managers and frontline practitioners. These questionnaires gathered quantitative and qualitative data related to organizational issues in the implementation of coordinated care and telehealth. Three analytical stages were followed: (1) preliminary analysis for a direct comparison of the responses of service managers and frontline staff to a range of organizational issues, (2) secondary analysis to establish statistically significant relationships between baseline and follow-up questionnaires, and (3) thematic analysis of free-text responses of service managers and frontline staff.

**Results:**

Both frontline practitioners and managers highlighted that training, tailored to the needs of different professional groups and staff grades, was a crucial element in the successful implementation of new services. Frontline staff were markedly less positive than managers in their views regarding the responsiveness of their organization and the pace of change.

**Conclusions:**

The data provide evidence that the setup of health care services is positively associated with outcomes in several areas, particularly tailored staff training, rewards for good service, staff satisfaction, and patient involvement.

## Introduction

Health care services face escalating demands in managing chronic conditions due to significant demographic changes as the population ages, along with increased levels of obesity and sedentary lifestyles [1,2]. One response to these increased pressures is to enhance the level of coordination and integration between different health/social care agencies and staff to improve patient care and maximize value for money [3]. Coordinated care has been defined as the deliberate organization of patient care between health and other services to facilitate the appropriate delivery of health care services [4].

Telehealth and telecare are assistive technologies that are increasingly used to support the coordinated care of patients with social care needs or chronic conditions such as diabetes and heart disease. With the advent of COVID-19, the vital role of telehealth in both health emergencies and usual health care delivery has been further highlighted. The use of remote triaging for care and video consultations for disease diagnosis now appear to be indispensable; however, the adoption of telehealth remains limited and variable [5]. These technologies enable exchanges of information between agencies, remote monitoring of health status, and have the potential to facilitate greater independence to ultimately reduce hospital visits and improve outcomes [6-8]. In particular, telehealth has the potential to support the close working of health and social care systems, which is particularly important for the management of elderly patients [9,10].

In reality, however, the potential benefits of integration are rarely achieved, and the delivery of care is often a “loosely coupled” system. The difficulties of achieving integration are due to a number of factors, including increased marketization, lack of managerial knowledge, shortage of care workers, and underfunded social care services [11]. Tuckson et al [12] noted the key trends that will influence the growth of telehealth care delivery, including: continuous innovation in consumer technology, continuous advancement in electronic health records and clinical decision support systems, projected shortages in the health professional workforce, and the growth of consumerism in health care.

As health services evolve to better manage patients using coordinated care approaches and monitoring technologies, staff are required to work in new ways and expand their roles. One key requirement for the success of these initiatives is the involvement of all staff so that they understand not only what is required of them but also that they consider their own stake in the system by having the opportunity to contribute their own ideas. At the same time, clinicians are expected not only to be engaged but also to lead throughout the process [13]. The need for engagement often provides a barrier to true coordination and integration. Although the fundamental importance of multidisciplinary teamwork is acknowledged [14,15], research suggests a “surprising lack of clarity” regarding purpose, objectives, leadership, and performance in many such teams [16]. A qualitative study of UK health professionals working in clinics, hospitals, pharmacies, and surgeries found that they were hampered by a lack of experience and concerns over losing face-to-face contact and missing key care information [17]. Efforts are being made to address these problems; for example, the Interprofessional Teamwork Innovation Model has been shown to facilitate a collaborative environment, enhance communication, and save time [18].

Other models have made explicit the link of good teamwork and effectiveness with innovation in health care delivery [19], and emphasized that engaged staff deliver a better experience for patients, have higher levels of morale, and make fewer errors [20]. To achieve this, health services are challenged to move from a model of “heroic leadership” [21] and traditional care delivery within “specialist silos” [22] to benefit from a positive relationship between shared leadership and team performance [23], with the goal of developing more integrated systems of care and a network of partnerships between services. The COVID-19 pandemic has forced health systems, hospitals, and clinics to implement telehealth rapidly; however, the challenge remains to support health care professionals in such a rapidly changing environment [24].

The current study was carried out as part of the Advancing Care Coordination and Telehealth (ACT) program [25,26], which investigated how health services are designed and configured to implement coordinated care and telehealth approaches. The ACT program explores the organizational and structural processes needed to implement coordinated care services on a large scale, and provides a foundation to overcome current barriers for extensive adoption of telehealth and coordinated care. Five European Union regions worked together to identify best practice in terms of scaling up coordinated care: Basque Country, Scotland, Lombardy, Groningen, and Catalonia. A key driver of the ACT program was to consider the attitudes, perceptions, and role of staff and their engagement in the deployment and configuration of services. Therefore, the key aim of the present study was to understand how the organizational setup influences the perceptions and experience of service managers and frontline staff during the development and deployment of integrated care with and without telehealth.

## Methods

### Study Design

Five diverse health services agreed to participate in the study, each of which differed in size and treated a wide range of health conditions. Most of these services were designed for older populations for the care of long-term chronic conditions, and incorporated either or both telehealth and coordinated care approaches. A convenience sampling approach was followed; to be included in the study, each service had to use either or both telehealth and coordinated care. To understand the content and structure of the health services under study, a three-stage mixed methodology was used. A quantitative, descriptive baseline questionnaire was completed by service managers to understand the structure and purpose of each European health service. This allowed for the development of a more targeted questionnaire, completed by both service managers and frontline staff, collecting both quantitative data and qualitative responses.

Questionnaires were developed iteratively and reviewed by health care representatives of each European region as part of regular meetings with the ACT program consortia. A concise set of questions was then developed to assess levels of engagement, perceptions, and experience of staff working within the specific health care contexts. Each question was rated on a Likert scale (strongly agree, agree, no opinion, disagree, strongly disagree), followed by open-ended questions allowing staff to describe their experiences in their own words. Topics included changes to daily activities, professional status, the role of telehealth and coordinated care in services, long-term vision, the role of feedback, and extent of training. Questionnaires were deployed using participants’ first language, with free-text responses translated prior to analysis.

### Analytical Approach

Three analytical stages were followed: (1) preliminary analysis allowing for a direct comparison of responses of service managers and frontline staff to a range of organizational issues, (2) analysis to establish statistically significant relationships between baseline and follow-up questionnaires; and (3) systematic examination of free-text responses of service managers and frontline staff using qualitative thematic analysis.

Statistical analysis was undertaken to investigate the relationship between baseline questionnaire responses that described the services and follow-up questions asked to frontline staff. The nonparametric Mann-Whitney *U* test was used to compare categorical (baseline) and continuous (follow-up questionnaire Likert scales) variables. Significance was set at *P*<.05. Only statistically significant results are reported in this paper. A thematic analysis of the follow-up questionnaire free-text responses was conducted to provide further insight into the findings. The three analytical stages allowed for gaining an overall understanding of each health service participating in the study, the degree to which service managers and frontline staff were in alignment with their views, and a more detailed examination of their views regarding key issues.

## Results

### Response Rates

Table 1 shows the response rates for service managers and frontline staff. The overall response rate was 78% for service managers and 47% for frontline staff, with the highest response rate for service managers and frontline staff combined from the Basque Country (88%), Spain, and the lowest from Groningen, Netherlands (55.5%).

**Table 1:** Response rates by regions and service level.

Program	Service Managers		Frontline Staff	
	Target	% Response rate	Target	% Response rate
Basque Country (Spain)	6	100	141	76
Catalonia (Spain)	7	57	35	80
Groningen (Netherlands)	4	100	161	11
Lombardy (Italy)	12	66	109	52
West-Lothian (Scotland)	3	100	29	44
All programs	32	78	475	47

### Staff Roles in Health Care Services

Table 2 summarizes the responses of frontline staff with respect to the importance of their role and change in their activities, categorized according to their own project managers’ responses to the baseline questions. Several factors were found to contribute significantly to frontline staff reporting that their importance within their organization had changed, including the tailoring of training (*P*<.001), existence of a strategy to share benefits (*P*=.02), regular evaluation of staff satisfaction of the service (*P*=.01), support for the service (*P*=.046), rewards for good service (*P*=.003), and involvement of patients in coordinated care (*P*=.04). The frontline staff statement describing daily activities changing significantly since the introduction of a service was positively associated with one service manager question: “Is the content and methods of training tailored to the needs of different professional groups and staff grades?” (*P*=.002).

The free-text responses illustrated how the roles of health professionals are changing. Staff described working with the same criteria, working in a coordinated manner, and using shared medical records, resulting in more efficient care: “To do more with less hours” [frontline staff, Groningen]. Giving more responsibility to different health professionals was linked to a sense of being valued and an increase in status. Staff perceived a decrease in hospital admissions and emergency room visits, thereby optimizing resources. An integrated service approach was viewed as facilitating enhanced control of chronic diseases; extra support by case managers; opportunity to provide more coherent, integral, and individual care; and increased monitoring. There was the possibility of earlier intervention and therefore more proactive treatment, “without having to go through the hoops of all specialties” [frontline staff, Catalonia]*.* Older adults were more empowered, reducing frailty, enabling people to live on their own for as long as possible:

In general I think that the program gives security to the professionals when making decisions; the patient feels more accompanied, improving his or her perception of quality of care and quality of life, and for the organization, it optimizes resources and improves efficiency, efficacy and care.

frontline staff, Catalonia

**Table 2:** Responses of frontline staff and service managers with respect to the role of staff in organization.^a^

Question			Yes (mean)	No (mean)	*P* value
**Frontline staff statement:** **My importance within the organization has been raised because of this service**					
	Are the content and methods of training tailored to the needs of different professional groups and staff grades (ie, senior/junior medical staff?)		3.59	3.07	<.001
	Is there a strategy to capture and share examples of benefits or helpful working practices?		3.28	3.70	.02
	Is staff satisfaction of your service evaluated regularly?		3.62	3.24	.01
	Has there been support for your service?		3.32	3.82	.046
	Are rewards given for good service?		4.25	3.32	.003
	Are patients involved in coordinated care?		3.39	3.06	.04
**Frontline staff statement: My day-to-day activities have changed significantly as a result of this service**					
	Are the content and methods of training tailored to the needs of different professional groups and staff grades (ie, senior/junior medical staff?)		3.82	3.47	.002

^a^Frontline staff responses are rated on a 5-point continuous Likert scale (1= strongly agree, 5=strongly disagree), which are presented according to the binary service manager response (yes/no).

Success in engaging staff and ensuring they felt valued entailed ensuring that messages reached all staff groups and that staff were able to give feedback regarding service development, enabling them to monitor progress and be party to the views of other stakeholders. Of particular importance, according to the free-text responses, was ensuring that the benefits of health care services were communicated and that technology was utilized to establish productive working relationships between different professional teams, for example using videoconferencing sessions between clinics.

### Staff Views of Organization: Organizational Responsiveness

Table 3 summarizes the frontline staff responses according to their own service managers’ responses to baseline questions regarding organizational change and organizational responsiveness. Several factors contributed significantly to frontline staff reporting that their organization had changed for the better as a result of the introduction of their service. These were tailored training according to staff need (*P*<.001), the use of a strategy to share examples of benefits and good practice (*P*=.045), regular staff evaluation of their service (*P*<.001), rewards for good service (*P*=.04), patient involvement in coordinated care (*P*=.02), a new role in their organization for coordinated care in their service (*P*=.004), and the existence of a business model to support the organizational structures involved in the provision of telehealth (*P*=.046). Two factors contributed significantly to frontline staff agreeing that their organization is responsive to feedback and change: tailored training according to staff experience and need (*P*<.001) and regular evaluation of staff satisfaction of their service (*P*<.001).

A perception of significant organizational change was reported in the free-text responses where health care services were considered to have adopted a patient-centered health care model and a shared decision-making approach, coupled with the decision to invest in a service. Thereafter, there had to be an “acceptance of the need to work in new ways and deliver care nearer to patients in their own home, expanded roles, skills of staff, and blurring of professional boundaries” [service manager, Scotland]*.* Expanded roles could also be seen elsewhere as an agent for change. For example, where general practitioners had “modified their way to manage chronic patients by proactive medicine” [service manager, Lombardy].

In terms of telehealth/telecare implementation, one service had adapted with the formation of a team to deal with requests for access to telecare into a wider “care at home service,” allowing a streamlined approach to support hospital discharge. Another service manager reported a “seamless” adoption due to additional services offered by general practitioner offices alongside telemedicine services, coupled with the “adequate training” of staff [service manager, Lombardy]. Elsewhere, change was observed in the ways in which staff work more than changes in the organization itself:

There is a paradigm shift in the care model that is catching on the staff. Although the organization still has some way to adjust the ways of working and evaluating their services to this shift in delivery. There is much work ahead.

service manager, Basque Country

It’s difficult to change in a short time the way to work, so it’s a long process that we’re doing but it’s not finished.

service manager, Lombardy

Change was perceived to be gradual, incremental, and iterative, and was also restricted by the nature of services. For example, with pilot programs, change is restricted due to a narrower focus, and a shorter timeframe and scale of a project.

**Table 3:** Frontline staff and service manager views of organization.^a^

Question			Yes (mean)	No (mean)	*P* value
**Frontline staff statement: The organization has changed for the better as a result of the program**					
	Are the content and methods of training tailored to the needs of different professional groups and staff grades (ie, senior/junior medical staff?)		3.87	3.43	.001
	Is there a strategy to capture and share examples of benefits or helpful working practices?		3.60	3.95	.045
	Is staff satisfaction of your service evaluated regularly?		4.09	3.50	<.001
	Are rewards given for good service?		4.25	3.62	.04
	Are patients involved in coordinated care?		3.72	3.37	.02
	Is there a new role or function in the organization for coordination of care in your service?		3.56	4.13	.004
	Is there a business model to support organizational structures involved in the provision of telehealth?		4.22	3.68	.046
**Frontline staff statement: The organization is responsive to feedback and changes occur quickly**					
	Is the content and methods of training tailored to the needs of different professional groups and staff grades (ie, senior/junior medical staff)?		3.47	2.97	.001
	Is staff satisfaction of your service evaluated regularly?		3.66	3.07	<.001

^a^Frontline staff responses are rated on a 5-point continuous Likert scale (1= strongly agree, 5=strongly disagree), which are presented according to the binary service manager response (yes/no).

### Organizational Commitment to Broadening Services

Table 4 summarizes the views of frontline staff regarding their organization incorporating telehealth and/or coordinated care, categorized according to their own service manager’s responses to baseline questions. The factors contributing significantly to frontline staff views were: tailored training for different staff groups (*P*=.006), rewards for good service (*P*=.004), patient involvement in coordinated care (*P*=.006), use of financial incentives to encourage telehealth adoption (*P*=.007), use of financial incentives for positive outcomes (*P*=.002), and adhering to a business model to facilitate financial alignment and incentives (*P*=.03).

In the free-text responses, frontline staff commented on how coordinated care approaches were benefitting their patients. They described how patients were more supported, such as when they are assigned a contact person (eg, a case manager) who guided them through the service. This improved their overall experience of care markedly:

The service is truly patient-centered and provided within the patient’s own home with their family and carers around them. This avoids the distress experienced during a hospital admission and the exposure to the risk of hospital acquired infection. The patients, when asked, express home treatment as their preference in most cases.

frontline staff, Scotland

When sufficiently trained and rewarded, staff were more likely to view coordinated care approaches as fostering relationships between different care providers such as nursing home staff, primary care, and hospitals, or better cooperation between general practitioners, district nurses, and elderly medicine specialists in order to provide better care:

To provide specialized care for people with complex needs by offering coordination with specialized care teams.

frontline staff, Catalonia

For myself, the most positive elements are that everyone involved are working together for the good of the service user and organization. Everyone is working as one

frontline staff, Scotland

Staff commented positively on the value of new technologies in health care, both for patients and frontline staff: “Being able to respond quickly to emergencies and [provide] reassurance for users” [frontline staff, Scotland]; “Telecare is adaptable and centered around the individual—it is less intrusive. It provides reassurance for individuals and their families” [frontline staff, Scotland].

Where there was also shown to be a strong business case, telehealth/telecare was seen to cut costs and reduce hospital visits.

**Table 4:** Frontline staff and service manager responses to organizational commitment.^a^

Question			Yes (mean)	No (mean)	*P* value
**Frontline staff statement:** **The organization wishes all appropriate clinical services to include telehealth and/or coordinated care**					
	Are the content and methods of training tailored to the needs of different professional groups and staff grades (ie, senior/junior medical staff?)		3.91	3.62	.006
	Are rewards given for good service?		4.44	3.37	.004
	Are patients involved in coordinated care?		3.82	3.52	.006
	Are there financial incentives to use telehealth?		3.46	4.11	.007
	Are financial incentives related to outcomes?		3.46	4.25	.002
	Is there a business model to facilitate financial alignment/ incentives		3.46	3.96	.03

^a^Frontline staff responses are rated on a 5-point continuous Likert scale (1= strongly agree, 5=strongly disagree), which are presented according to the binary service manager response (yes/no).

### Future Role of Organization

Table 5 summarizes the frontline staff responses to three questions regarding the future role of the organization categorized according to their own service managers’ responses to baseline questions. Two factors contributed significantly to frontline staff reporting that telehealth/coordinated care was an important aspect of future initiatives to improve health care delivery: use of rewards for good service (*P*=.05) and the involvement of patients in coordinated care (*P*<.001). Three factors contributed significantly to frontline staff agreeing that there is recognition that the approach of their service is the future direction for their organization: the use of tailored training (*P*<.001), the existence of a guideline or protocol (*P*=.047), and barriers to implementing the service (*P*<.001). Two factors contributed significantly to frontline staff reporting that everyone recognizes that the approach of their service will bring long-term benefits: tailored training for different professional groups (*P*<.002) and regular evaluation of awareness of their service amongst staff (*P*=.046).

**Table 5:** Frontline staff and service manager responses to the future role of organization.^a^

Question			Yes	No	*P* value
**Frontline staff statement: Telehealth/coordinated care is an important aspect of future initiatives to improve care delivery**					
	Are rewards given for good service?		4.56	4.11	.05
	Are patients involved in coordinated care?		4.24	3.67	<.001
**Frontline staff statement: Everyone recognizes that the approach of this service is the future direction of the organization**					
	Are the content and methods of training tailored to the needs of different professional groups and staff grades (ie, senior/junior medical staff?)		3.78	3.36	.001
	Is there a guideline or protocol?		3.51	3.81	.047
	Were there any barriers to implementing the service?		3.58	3.57	<.001
**Frontline staff statement: Everyone recognizes that the approach of this service will bring long-term benefits**					
	Are the content and methods of training tailored to the needs of different professional groups and staff grades (ie, senior/junior medical staff?)		3.83	3.44	.002
	Is staff awareness of your service evaluated regularly?		3.73	3.43	.046

^a^Frontline staff responses are rated on a 5-point continuous Likert scale (1= strongly agree, 5=strongly disagree), which are presented according to the binary service manager response (yes/no).

The free-text responses suggest that all service managers viewed their service(s) as fitting in with the broader health care aims and objectives of their organization, such as having electronic patient records for the whole population, or in a more overarching sense: “it enables the integration and coordination among care delivery levels in chronic diseases like heart failure” [service manager, Basque Country]; “Yes it does totally fit with the strategic plan, the social accountability, the mission and values of the organization” [service manager, Catalonia].

When asked about any negative aspects of their service, some service managers reported none, but others noted that a rush to implementation did not take into account the time required to introduce new initiatives and to ensure the involvement of a large number of professionals.

### Difference in Views Between Frontline Staff and Service Managers

Both service managers and frontline staff answered the same follow-up questionnaire statements discussed in the previous sections, allowing for a direct comparison of their views. Service managers held uniformly more positive views than frontline staff. As expected, the latter were more likely to report a significant change in their day-to-day activities when compared to the service managers. However, while the majority of service managers considered their organization to be supporting the implementation of telehealth, a significant proportion of frontline staff held no opinion, and a minority disagreed or strongly disagreed with the statement.

Service managers were more likely to view their organization as supporting coordinated care implementation, that their organization had changed as a result of a new service, that their organization wishes all clinical services to include telehealth/coordinated care, and that the approach of the service would bring long-term benefits and was the future direction of the organization. Frontline staff were markedly less positive in their views regarding the responsiveness of their organization and pace of change. Service managers and frontline staff were closer in agreement that their organization was training all staff in the implementation of a service, which was conspicuous as the single area in which both staff and managers held similar views.

## Discussion

### Pivotal Role of Staff Training

The quantitative data presented provides evidence that telehealth technologies are becoming increasingly embedded in frontline services. Participants considered how telehealth reassured patients and provided the opportunity for health professionals to intervene in real time, act quickly, and provide flexible care. Similarly, study participants suggest that coordinated care approaches are increasingly becoming part of daily practice. They perceived that coordinated care provided enhanced control of chronic disease and the opportunity to offer more coherent, integral, supportive, and individualized care. The findings also supported the view that coordinated care fosters relationships between different care providers such as nursing home staff, primary care hospitals, or better cooperation between the general practitioner, district nurse, and elderly medicine specialist to offer better care.

The findings clearly emphasized the importance of training, and in particular training tailored to the needs of different professional groups and staff grades involved in coordinated care or telehealth. Tailoring training was positively associated with favorable perceptions of professional status, changes in daily activities, the view that a health care organization had changed for the better, agreement that an organization wants all clinical services to include telehealth or coordinated care, and agreement that a service is the future direction of an organization. All regions reported that training had been provided to equip staff with the knowledge and skills required to deliver their coordinated care or telehealth services. Topics covered in the training varied as much as the services themselves; however, training was not always tailored to the needs of different professional groups and staff grades. To maximize benefit, training should be provided as part of telehealth and coordinated care implementation; be practical, purposeful, and timely; and encourage a patient-centered approach to foster positive patient-health care professional relationships [27].

Previous research shows that staff require not only access to appropriate resources and adequate staffing levels [16], but also an organization that supports personal development [28] and provides training aligned to staff roles to fulfill the demands of working within multidisciplinary health care teams [29]. Such support is not simply part of an initial roll out but rather an ongoing dialog between frontline professionals and managers [30] in health care settings actively promoting a learning climate [31]. Most services in this study reported that staff awareness was evaluated regularly and that those findings were acted upon.

Rewards for good service, staff satisfaction, and patient involvement in coordinated care featured prominently in this study. The former two could arguably be linked to staff training and continuing professional development. The latter is perhaps indicative of the need for all involved in the care process, including patients, to be included in the successful evolution of health services. Of note was the finding that the views of service managers were markedly more positive than those of frontline staff, suggesting that the positive messages conveyed from the top do not necessarily resonate with those working in the services. Perhaps unsurprisingly, all service managers viewed their service as fitting in with the broader health care aims and objectives of their organization, whereas frontline staff showed more variation in their responses. For effective implementation of coordinated care and telehealth, the views of these two groups of staff need to be in better alignment.

Significant perception of change was reported where health care services adopted a patient-centered health care model and a shared decision-making approach, coupled with the decision to invest in a service. Staff reported how their working practices had changed as a result of the introduction of new services. Such change was more likely to be evident among frontline staff if they had received tailored training. Change was also reported in contexts for which professionals have expanded or adapted their roles*.* However, the capacity for change could be restricted by the type of service, particularly smaller-scale pilots.

The qualitative, free-text responses highlighted how the roles of health professionals are changing, particularly with the use of coordinated care approaches and telehealth integrated into care pathways to optimize resources. These approaches were considered to allow for earlier intervention, proactive treatment, and independent living. Part of the considerable, but gradual, organization change, according to the responses, was the move to patient-centered health care with staff working in complex, multidisciplinary teams. This required an acceptance of the need to work together in new, innovative ways. Overall, staff viewed their services, which incorporate telehealth and coordinated care, as fitting in with the broader health care aims of their organization.

### Limitations

Both the types and the sizes of services analyzed in this study varied considerably, raising the question as to whether the integration of the data was appropriate. The current approach attempted to obtain a broad view with an intention of identifying overarching trends. Thus, it is not surprising that both service size and type were diverse, as these are Europe-wide real-life examples of the practice of integrative care. Furthermore, several services showed low response rates (Table 1), but these were nevertheless included so as to provide a comprehensive view of the range of services studied. This does produce some statistical weaknesses to the study.

The baseline and service manager questionnaires were used for descriptive analysis due to the low number of responses. The one service manager per service structure also meant that the service managers were identifiable, allowing for the possibility of respondents vetting their answers and considering the implications of making negative comments, thus resulting in bias. The baseline questionnaire was used to group (classify) the various services allowing for statistical comparisons on (intermediate) outcomes across different programs. For statistical analysis, only nonparametric testing (Mann-Whitney *U* test) was used.

Data were collected at a specific point in time, allowing for only a snapshot of experience. However, attempts were made to triangulate the different types of data collected at different time points. Limitations with terminology were also identified as part of a large pan-European study where it was assumed that terms to describe health services conveyed similar meanings across different cultural contexts. The analysis of free-text responses collected from frontline staff and service managers provide insight into the views of professionals working in the various services under study; however, it should not be considered as a comprehensive overview of the spectrum of views from all staff from all services. More in-depth qualitative studies featuring health care professionals are needed. A longitudinal study exploring the experiences of nurses and healthcare staff reported that the changes instigated due to telehealth implementation elicited a sense of threat. Such experiences, captured over time, would be unlikely to be detected in a study of this nature [32].

### Evolving Services, Evolving Roles

This analysis presents a snapshot of a diverse range of European health services attesting to use coordinated care or telehealth. It also provides new insight into the challenges faced by health care organizations in terms of embracing change, integrating new technologies, and fostering closer relations between health and social services in order to meet the challenge of caring for aging populations. This then places the onus to change on staff, particularly those on the frontline. Those at the “sharp end,” who are often most aware of how problems can be addressed, can feel “powerless to bring about change” [16]. Nevertheless, health care interventions have demonstrated how small changes can generate significant improvement for patients, staff, and hospital performance [33]. Increased availability and visibility of specific health care professionals and closer working relationships between different professional groups are required, in addition to conferring greater responsibility on those required to coordinate specialist primary care teams and to case manage patients through myriad health and social services, which would help to ensure the continuity of care and appropriate utilization of resources.

Such change, both in terms of routine and emergency care [5], demands the refinement of decision-making protocols in complex care situations and defining the specific levels and intensity of care needed. Continuous feedback is required between professionals, service operators, and project teams to ensure that evolving needs are met and that change is made incrementally in addition to active participation by multidisciplinary teams in the planning, coordination, and modes of data collection within their service. For example, this may include the use of specialist discharge planners to ensure patients transit appropriately through care pathways [34], or a dedicated fund to forge integrated working of health and social care teams [13] as part of whole-systems change.

Although telehealth presents exciting opportunities for patients, carers, and health professionals, this fast-moving field demands the constant evolution and adaptation by all involved actors. As telehealth becomes more embedded and intrinsically linked with care and service delivery, so must the quality of evidence to ensure that clinicians can confidently make decisions that patients and carers will trust and adhere to [12]. This can help health services to adapt when telehealth and coordinated care approaches are most needed as we face the challenges of COVID-19 [5,24]. Staff increasingly accept the need to work in new ways and deliver care beyond conventional care settings and within patients’ homes. They are working within and between ever more fluid boundaries of health and social care; within this context, the need for specialized training tailored to staff needs is increasingly salient. This requires organizational commitment to training and development of clinical staff, not only in leadership [20] but at all levels. This will ensure that a diverse range of professional groups can deliver the highest levels of innovation in patient care [28], and consequently improve the quality, safety, and effectiveness of health care delivery [31].

## Abbreviations

ACT: Advancing Care Coordination and Telehealth

## References

[1] Christensen K, Doblhammer G, Rau R, Vaupel JW (2009). Ageing populations: the challenges ahead. Lancet.

[2] Wang YC, McPherson K, Marsh T, Gortmaker SL, Brown M (2011). Health and economic burden of the projected obesity trends in the USA and the UK. Lancet.

[3] Wagner EH, Groves T (2002). Care for chronic diseases. BMJ.

[4] McDonald K, Sundaram V, Bravata D, Lewis R, Lin N, Kraft S, McKinnon M, Paguntalan H, Owens D (2007). Closing the Quality Gap: A Critical Analysis of Quality Improvement Strategies. Technical Review AHQR Publication No. 04(07)-0051-7.

[5] Smith AC, Thomas E, Snoswell CL, Haydon H, Mehrotra A, Clemensen J, Caffery LJ (2020). Telehealth for global emergencies: Implications for coronavirus disease 2019 (COVID-19). J Telemed Telecare.

[6] Steventon A, Bardsley M, Billings J, Dixon J, Doll H, Hirani S, Cartwright M, Rixon L, Knapp M, Henderson C, Rogers A, Fitzpatrick R, Hendy J, Newman S, Whole System Demonstrator Evaluation Team (2012). Effect of telehealth on use of secondary care and mortality: findings from the Whole System Demonstrator cluster randomised trial. BMJ.

[7] Inglis SC, Clark RA, McAlister FA, Ball J, Lewinter C, Cullington D, Stewart S, Cleland JG (2010). Structured telephone support or telemonitoring programmes for patients with chronic heart failure. Cochrane Database Syst Rev.

[8] McLean S, Nurmatov U, Liu JLY, Pagliari C, Car J, Sheikh A (2012). Telehealthcare for chronic obstructive pulmonary disease: Cochrane Review and meta-analysis. Br J Gen Pract.

[9] Bennet G, Ebrahim S (1992). The essentials of health care of the elderly.

[10] Gröne O, Garcia-Barbero M, WHO European Office for Integrated Health Care Services (2001). Integrated care: a position paper of the WHO European Office for Integrated Health Care Services. Int J Integr Care.

[11] Leichsenring K (2004). Developing integrated health and social care services for older persons in Europe. Int J Integr Care.

[12] Tuckson RV, Edmunds M, Hodgkins ML (2017). Telehealth. N Engl J Med.

[13] Appleby J, Galea A, Murrary R (2014). The NHS productivity challenge: Experience from the front line.

[14] Leonard M, Graham S, Bonacum D (2004). The human factor: the critical importance of effective teamwork and communication in providing safe care. Qual Saf Health Care.

[15] Nembhard IM, Edmondson AC (2006). Making it safe: the effects of leader inclusiveness and professional status on psychological safety and improvement efforts in health care teams. J Organiz Behav.

[16] Dixon-Woods M, Baker R, Charles K, Dawson J, Jerzembek G, Martin G, McCarthy I, McKee L, Minion J, Ozieranski P, Willars J, Wilkie P, West M (2014). Culture and behaviour in the English National Health Service: overview of lessons from a large multimethod study. BMJ Qual Saf.

[17] Kayyali R, Hesso I, Mahdi A, Hamzat O, Adu A, Gebara SN (2017). Telehealth: misconceptions and experiences of healthcare professionals in England. Int J Pharm Pract.

[18] Li J, Talari P, Kelly A, Latham B, Dotson S, Manning K, Thornsberry L, Swartz C, Williams MV (2018). Interprofessional Teamwork Innovation Model (ITIM) to promote communication and patient-centred, coordinated care. BMJ Qual Saf.

[19] Borrill C, West M, Shapiro D, Rees A (2000). Team working and effectiveness in health care. Br J Health Care Manag.

[20] Ham C (2012). Leadership and engagement for improvement in the NHS: Together we can.

[21] Ham C, Baker GR, Docherty J, Hockey P, Lobley K, Tugendhat L, Walshe K (2011). The future of leadership and management in the NHS: no more heroes.

[22] Allen KR, Hazelett SE, Radwany S, Ertle D, Fosnight SM, Moore PS (2012). The Promoting Effective Advance Care for Elders (PEACE) randomized pilot study: theoretical framework and study design. Popul Health Manag.

[23] D’Innocenzo L, Mathieu JE, Kukenberger MR (2016). A Meta-Analysis of Different Forms of Shared Leadership–Team Performance Relations. J Management.

[24] Wosik J, Fudim M, Cameron B, Gellad ZF, Cho A, Phinney D, Curtis S, Roman M, Poon EG, Ferranti J, Katz JN, Tcheng J (2020). Telehealth transformation: COVID-19 and the rise of virtual care. J Am Med Inform Assoc.

[25] Dueñas-Espín I, Vela E, Pauws S, Bescos C, Cano I, Cleries M, Contel JC, de Manuel Keenoy E, Garcia-Aymerich J, Gomez-Cabrero D, Kaye R, Lahr MMH, Lluch-Ariet M, Moharra M, Monterde D, Mora J, Nalin M, Pavlickova A, Piera J, Ponce S, Santaeugenia S, Schonenberg H, Störk S, Tegner J, Velickovski F, Westerteicher C, Roca J (2016). Proposals for enhanced health risk assessment and stratification in an integrated care scenario. BMJ Open.

[26] (2019). https://www.philips.com/a-w/about/news/archive/standard/news/press/2019/20190402-philips-presents-results-from-3-year-telehealth-program-impacting-over-100000-patients-across-europe.html.

[27] Guise V, Wiig S (2017). Perceptions of telecare training needs in home healthcare services: a focus group study. BMC Health Serv Res.

[28] West M, Dawson J, Admasachew L, Topakas A (2011). NHS Staff Management and Health Service Quality: Results from the NHS Staff Survey and Related Data.

[29] Mohr JJ, Batalden PB (2002). Improving safety on the front lines: the role of clinical microsystems. Qual Saf Health Care.

[30] Parker LE, Kirchner JE, Bonner LM, Fickel JJ, Ritchie MJ, Simons CE, Yano EM (2009). Creating a quality-improvement dialogue: utilizing knowledge from frontline staff, managers, and experts to foster health care quality improvement. Qual Health Res.

[31] Salas E, Almeida SA, Salisbury M, King H, Lazzara EH, Lyons R, Wilson KA, Almeida PA, McQuillan R (2009). What Are the Critical Success Factors for Team Training in Health Care?. Jt Comm J Qual Saf.

[32] Sharma U, Clarke M (2014). Nurses' and community support workers' experience of telehealth: a longitudinal case study. BMC Health Serv Res.

[33] Moore C, Buchanan DA (2013). Sweat the small stuff: a case study of small-scale change processes and consequences in acute care. Health Serv Manage Res.

[34] Ham C (2005). Lost in Translation? Health Systems in the US and the UK. Soc Policy Adm.

